# Advancement in Understanding Immune Responses against Zoonotic Infections

**DOI:** 10.3390/tropicalmed8060305

**Published:** 2023-06-02

**Authors:** Yuanyue Tang, Zhongyi Jiang, Qiuchun Li

**Affiliations:** 1 Key Laboratory of Prevention and Control of Biological Hazard Factors (Animal Origin) for Agri-food Safety and Quality, Ministry of Agriculture of China, Yangzhou University, Yangzhou 225009, China; tangyy@yzu.edu.cn (Y.T.); mx120200990@stu.yzu.edu.cn (Z.J.); 2 Jiangsu Key Laboratory of Zoonosis/Jiangsu Co-Innovation Center for Prevention and Control of Important Animal Infectious Diseases and Zoonoses, Yangzhou University, Yangzhou 225009, China; 3 Joint International Research Laboratory of Agriculture and Agri-Product Safety, Yangzhou University, Yangzhou 225009, China

This Special Issue focuses on the recent advancements in our understanding of immune responses against zoonoses, which include viral, bacterial, parasitic and fungal diseases. It is estimated that approximately 61% of human infections are caused by zoonoses, among which over 70% originated from wildlife species [1,2]. Zoonoses present a serious threat to public health, and it is crucial for the governments to take measures to prevent and control zoonotic infection. Understanding the immune responses induced by these pathogens is critical for us to develop measures to control zoonotic diseases. In this Special Issue, there are five papers published upon peer review acceptance, including one editorial and four research papers. The research papers contribute to a better understanding of immune responses in zoonotic infections.

The contributions of these research papers can be summarized as follows: The first paper focuses on using a protein calreticulin from *Echinococcus multilocularis* (*Em*CRT) as an immunogen to induce a protective immune response in BALB/c mice, with the aim of finding a vaccine to prevent *E. multilocularis* infection. The *Em*CRT protein is an ubiquitous protein with Ca^2+^-binding ability [3]. Recombinant *Em*CRT protein was constructed and expressed in a prokaryotic system, and then injected with Freund’s adjuvant into mice. The results showed that the levels of IgG, IgG1, and IgG2a antibodies in the immunized mice were significantly increased, and the mice also exhibited a certain protective efficiency, which can resist *E. multilocularis* infections. This study suggests that E*m*CRT protein is an important immunogen and can be further developed as a potential vaccine against *E. multilocularis* infection [4]. The second paper also focuses on the immune response caused by *Em*CRT protein. These further experiments showed that *Em*CRT can bind to human complement C1q and inhibit its binding to IgM, thereby reducing the activation of immune cell receptors and the occurrence of immune attack. Furthermore, *Em*CRT was found to suppress the chemotactic effect of C1q on human mast cells. These findings suggest that *E. multilocularis* uses *Em*CRT to interfere with the host immune attack process, which may be a strategy of immune evasion. This study further indicates that *Em*CRT could be developed as a vaccine candidate against *E. multilocularis* infection [5]. The third study aimed to investigate the maternal-fetal immune transfer of Nipah virus [6]. Although passive protection through immune transfer from mothers has been reported in diseases such as pertussis and influenza A [7], it has not been detected in Nipah virus infections. Through the study of a couple infected with Nipah virus in Bangladesh, it was found that maternal antibodies against Nipah virus could be transmitted to the fetus through the placenta, which is the first evidence of the vertical transmission of immunity in Nipah virus infection. This study also highlights the prevalence of Nipah virus, its effects on humans, and current preventive and treatment methods. The results of this study can be used as a reference for the further exploration of the possibility of Nipah-specific antibodies transferring through the placenta, their potential to protect newborns, and how they affect vaccine recommendations. The fourth study aimed to investigate the response of mouse dendritic cells (DCs) to BCG and explore the immune effects of different subtypes of DCs [8]. Tuberculosis (TB) caused by *Mycobacterium tuberculosis* (MTB) is reported to cause an estimated 8 million new cases and 1.3 million deaths every year [9]. Therefore, it is crucial to understand the immune responses of MTB infections. The results showed that the uptake rate of BCG and the number of intracellular bacteria in splenic pDC were significantly higher than those in cDC, CD8^+^ and CD8 cDC subtypes. However, during BCG infection, the expression levels of CD40, CD80, CD86 and MHC-II molecules in splenic cDC and CD8 cDC subtypes were significantly up-regulated compared with those in pDC. Splenic cDC and pDC were widely involved in the immune response of mice to BCG infection. pDC had a higher uptake of BCG and cDC induced stronger immune effects. This discrepancy of DCs could help us to find potential vaccine targets against TB.

The papers covered aspects of immune responses to zoonotic infections. These new findings contribute to a much better understanding of the immunology of these zoonotic infections. We take this opportunity to appreciate the willingness of all the authors to share their knowledge with international audiences.
